# Side Oscillation Milling: Modeling, Analysis, and Compensation of Cutting Forces Through Feed Optimization

**DOI:** 10.3390/ma18163789

**Published:** 2025-08-12

**Authors:** Michał Gdula, Piotr Żurek

**Affiliations:** Department of Manufacturing Techniques and Automation, Rzeszow University of Technology, al. Powstańców Warszawy 12, 35-959 Rzeszow, Poland; p_zurek@prz.edu.pl

**Keywords:** side milling, oscillation machining, cutting forces, feed optimization, hardened steel

## Abstract

This article presents an analysis and the modeling of cutting forces in the process of oscillation milling of side surfaces of workpieces made of hardened steel. In addition, the impact of the oscillation machining method on cutting forces was analyzed, taking into account feed optimization. A sinusoidal function was used to describe the trajectory of the tool in order to induce the oscillatory motion. The study is based on a set of 34 cutting tests using four end-mill cutters, each characterized by a unique combination of feed rate and sinusoidal downward and upward angles. This constitutes a novel approach to sine wave period selection. Empirical mathematical models of the cutting forces were developed using the response surface method. The results demonstrate that the sinusoidal trajectory of the tool movement, together with optimization of the feed rate, leads to a reduction in fluctuations and the stabilization of cutting forces, and an approximately 30% increase in the efficiency of this machining process.

## 1. Introduction

Scientific research has identified the cutting speed, feed rate, and tool movement trajectory pattern as the main process parameters that influence cutting forces [1,2,3,4,5]. This situation occurs during the finishing of side surfaces, including objects made of hardened steel. When hardened steel is machined, the role of the coolant is insignificant. This is dry machining, as very high temperatures (around 800–1000 °C) are generated in the cutting zone. Sudden cooling of the cutting edge by coolant can cause rapid temperature changes, resulting in microcracks and faster tool wear, especially for carbide tools. Furthermore, due to the high cutting speeds, the effectiveness of lubrication and cooling is significantly reduced [6,7]. The parameters determining the stereometry of the tool, i.e., the rake angle, the clearance angle, the corner radius, and the edge chamfer, influence the direction and magnitude of the cutting forces [8,9,10,11,12,13]. The possibility of changing their values and adapting them during the process is also severely limited or even impossible. However, a tool with variable pitch can be used to improve the cutting process. In addition, it is possible to indirectly influence the cutting mechanics by introducing an oscillatory (e.g., sinusoidal) tool movement trajectory during the milling process [14,15]. Guo et al. [16] presented a continuous oscillatory milling strategy intended to improve the uniformity of cutting edge wear through dynamic adjustment of the tool axis oscillation angle, thereby increasing tool longevity. They proved that this approach notably extended the tool life. Lopes da Silva et al. [17] introduced a methodology based on driven and self-propelled rotary tool cutting operations, employing tool path strategies in side milling with a solid end mill to modify the process kinematics. It was shown that both triangular and sinusoidal tool paths can enhance the effective feed in the direction perpendicular to the programmed feed. Nevertheless, it was also confirmed that the maximum thickness of material removed remains unchanged. As a result, this feed increase leads to a reduction in specific cutting force, causing the forces to be lower than those observed in the linear path. The results confirmed the effectiveness of alternative tool paths, such as sinusoidal and triangular, in improving surface roughness compared to the linear path. The study indicated a 20–30% decrease in surface roughness when these alternative paths were employed. However, its impact has not yet been clearly or conclusively established by the scientific community [17]. The pattern of the tool’s movement trajectory is of great importance. In some cases, a conventional motion trajectory can increase machining efficiency at the expense of shorter tool life [18,19,20], while unconventional cutting can have a positive effect on reducing cutting forces while utilizing the entire length of the cutting edge [17]. The trajectory of the tool also affects the method and efficiency of the chip and heat dissipation [21,22,23,24], as well as the dimensional accuracy and the surface quality [25]. In this aspect, Zhang et al. [26] proposed a method for optimizing tool paths and cutting parameters based on the CAM STEP-NC cloud. This reduced costs by 70% and improved efficiency by 70%. Stejskal et al. [27] proposed a methodology for the optimization of tool axis orientations in multi-axis milling toolpaths. The method is predicated on the calculation of two variables: the actual cutting diameter and the cutting speed during the toolpath. The improvement in surface quality and increase in productivity that ensues is the result of these calculations. Darzi et al. [28] developed a finite element model (FEM) for tool deflection, considering the variation in tool position resulting from forces exerted at the contact point with the workpiece. The purpose of this process is to improve the accuracy of the simulation in relation to experimental findings. A study was conducted in which the effects resulting from the stress state change and strain accumulation during DSIF were analyzed in relation to single-pass and reforming toolpaths. The results suggest that material points along the pyramid wall predominantly undergo deformation due to a combination of plane strain tension, plane strain compression, and shear. The exact deformation mechanism is governed by the position of the tools on the sheet surfaces relative to the material point under consideration. In their seminal paper, Zhang et al. [29] presented a comprehensive theoretical and experimental investigation into the generation of surface profiles of spiral toolpaths in the context of axial ultrasonic vibration-assisted polishing. A prediction calculation model for the generated surface profile of spiral toolpaths was also proposed, making a significant contribution to the field. In comparison with established methodologies, the present technique has the capacity to offer more realistic and precise depictions of the actual surface profile characteristics. Consequently, both superior and high-quality surfaces can be obtained. Chen et al. [30] proposed a method of optimization which was employed to generate a toolpath with six Degrees of Freedom for robotic flank milling. A sequential quadratic programming algorithm was proposed as a solution to this highly nonlinear problem, with the lexicographic order of arrays serving as the basis for the proposed algorithm. The simulations and experiments demonstrate that the proposed method has superior efficiency, robustness, and effectiveness in comparison with existing methods.

A number of research tasks for this study were carried out by a team led by Luis Norberto de Lacalle [31]. The most important ones are listed below. In the study by Perez-Ruis et al. [32], an oblique cutting Taylor-based model was proposed with the aim of quantifying the crystallographic effects on shear strength. In order to achieve this objective, the tool geometry, tool position, and laser scanning strategy were given full consideration, in addition to the microstructures, crystallographic textures, and grain morphologies of two samples with different layer thicknesses. This was achieved by means of scanning electron microscopy and electron backscatter diffraction. A significant correlation was identified between the fluctuations in cutting force and the predicted Taylor factor. Lopez De Lacalle et al. [33] presented a new methodology for the selection of the milling toolpaths on complex surfaces that minimize dimensional errors due to tool defection. They showed that, by applying this approach, in three axes, milling dimensional errors fall down from 30 mm to below 4 mm. In five axes milling errors can be kept below 15 mm in most of the cases.

There are many models available that use computer simulations and artificial intelligence methods to predict cutting forces [34,35,36,37,38,39]. However, in terms of unconventional manufacturing techniques and tool movement trajectories, such as sinusoidal ones, these models are insufficient for developing a rigorous mathematical and physical representation of the process. As a result, traditional experimental methods continue to remain widely used in research [40,41]. A comprehensive review of the literature revealed a research gap, as no studies have been conducted to evaluate the effect of the cutter’s upward and downward angles along a sinusoidal trajectory on the cutting forces during side milling operations on hardened steel. Furthermore, no studies were found concerning the rational selection of feed rates, taking into account the nonlinearity of the toolpath and the above-mentioned influence of its angles on machining dynamics.

Therefore, this study aims to at least partially fill this research gap by analyzing the tool’s sinusoidal ascent and descent angles, which directly determine the period of its trajectory, on the cutting forces. Based on the results obtained, the feed rate was optimized according to the specified angles along the sinusoidal trajectory. In addition, empirical models were formulated using response surface methodology (RSM).

The novelty of this work is

- The proposal of a sinusoidal tool path in the process of side milling of hardened steel and the development of a relationship that optimizes the feed rate on this path relative to commonly programmed linear paths;
- In addition, a method for determining the period of the sinusoidal path based on the angle of the surface shaping line of the workpiece has been proposed and developed.

## 2. Materials and Methods

**Figure 1:**
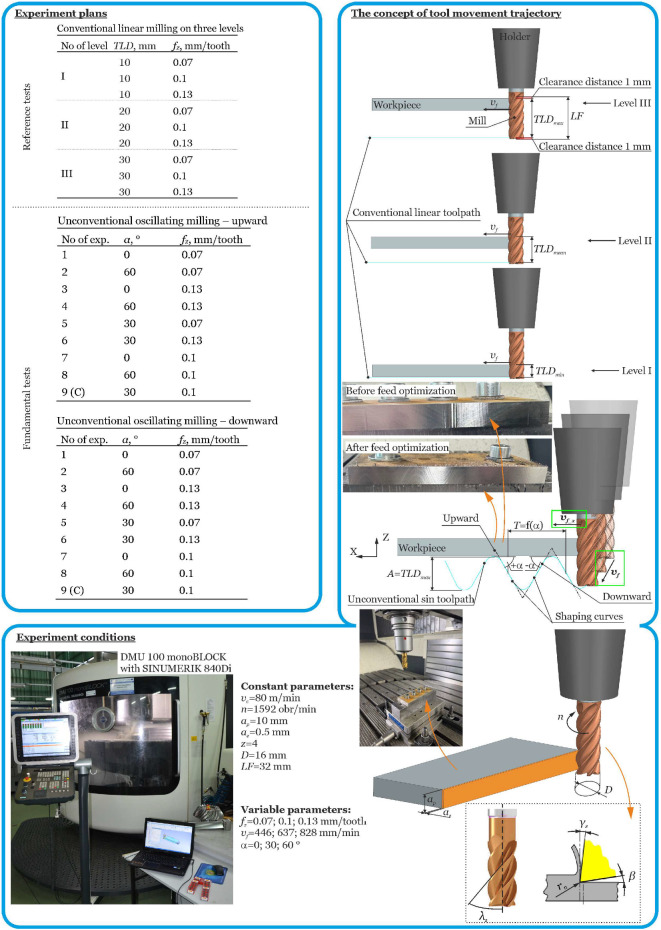
Technological conditions of the tests.

The study used 41Cr4 steel in a hardened state (53 HRC), a material commonly used for heavily loaded machine components. The test samples were rectangular prisms, each measuring 115 × 50 × 10 mm. Four Monster Mill 52784163 cutting tools, manufactured by CERATIZIT (CERATIZIT S.A. 101, Route de Holzem, 8232 Mamer, Luxembourg), were used, with a functional working length *LF* of 32 mm, a nominal diameter *D* = 16 mm, number of teeth *z* = 4, helix angle *λ_s_* = 40°, relief angle *γ_s_* = 10°, clearance angle *β* = 10°, and corner radius *r_n_* = 40 µm (see Figure 1). This allowed for reference conventional cutting tests with a linear motion trajectory at three levels measured from the cutter’s face surface. Furthermore, this allowed us to determine the amplitude of the unconventional sinusoidal trajectory of movement of the tool with an amplitude *A = TLD_max_* = 30 mm. The *TLD* parameter is the Tool Level Distance for a linear tool path (see Figure 1). When determining the test conditions, it was assumed that the maximum possible functional length of the tool would be used during the cutting process, taking into account the machine tool axis dynamics (acceleration and deceleration errors).

This results in three specific levels of linear motion trajectory, as well as a defined amplitude for the sinusoidal motion trajectory. The oscillation of the tool, whose amplitude was equal to the height of the working part of the tool and whose period resulted from the angle of inclination of the sine curve forming the machined surface, was performed in the Z-axis of the machine tool coordinate system. The research plans and technological test conditions are shown in Figure 1.

The experimental tests were conducted using the Design of Experiments (DOE) technique, employing a central composite circumscribed research plan with two levels and two factors. After each cutting test, the condition of the cutting edge was checked in terms of tool wear. No tool wear was observed. A measuring track consisting of a platform and a Kistler 9257B dynamometer (Kistler Group, Eulachstrasse 22, 8408 Winterhur, Switzerland), together with an NI measurement card and NI DAQ SignalExpress software (version number: 970f0), was used to measure the cutting forces. An extensive set of 34 cutting force measurements (102 counting each component separately) was performed, each of which was recorded three times. For each cutting force, a statistical analysis of variance ANOVA was performed with a 95% confidence level, using Student’s *t*-test.

The technological parameters of milling were determined by employing the response surface methodology (RSM) within the framework of a central composite rotatable design, a methodology implemented in Statistica 13.3 software.

The statistical method of determining the response surface, otherwise referred to as RSM, was employed to model the relationships between the cutting process parameters and the cutting force components. Research plans that utilize RSM methodology are concerned with the determination of a response surface based on a general equation, Equation (1), of the form

(1) $$Y=\beta_{0}+\beta_{1}X_{1}+\beta_{2}X_{2}+{\beta_{11}(X_{1})}^{2}+{\beta_{22}(X_{2})}^{2}+\beta_{12}X_{1}X_{2}$$

Consequently, a model is fitted to the experimental output values in order to capture the influence of the main input values (*X*_1_, *X*_2_), the interactions between the input values (*X*_1_*X*_2_), and the quadratic terms (*X*_12_, *X*_22_). The intercept of the arithmetic means of all the quantitative outcomes is referred to as *β*_0_. The parameters *β*_1_, *β*_2_, *β*_11_, *β*_22_, and *β*_12_ represent the coefficients derived from the experimental values of *Y*. In order to identify the most influential factors, create a suitable mathematical model, and evaluate its fit to the measurement data, regression statistics commonly employed in mechanical engineering, such as ANOVA, were utilized.

The experiments were conducted with a constant depth of cut set at 10 mm and width of cut set at 0.5 mm. Variable feed per tooth *f_z_* (*v_f_*) (feed rate), angle of shaping curves *α* (tool trajectory, see Figure 1), and tool work length distance *TLD* were used in the tests.

**Figure 2:**
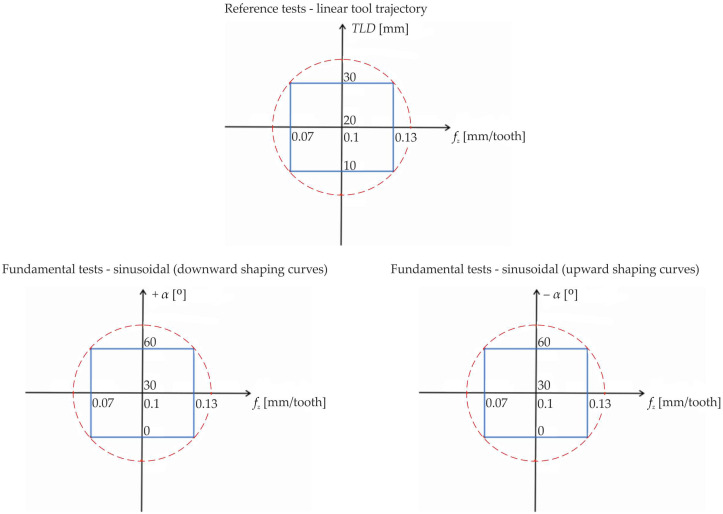
Schematic views of a central compositional design without star points.

The aim of the experiments was to determine the response surface using a central compositional design without star points (shown in Figure 2).

It should be noted that the machining tests for the sinusoidal motion trajectory were conducted in two stages. In the first stage, it was assumed that the feed rate *v_f_* was defined in a generally accepted manner, that is, the velocity vector was aligned with the tool movement direction. This allowed us to refer to tests conducted for the conventional linear trajectory of tool movement. In the second stage, the feed rate *v_f_* was optimized using the authors’ Equation (2) so that the constant feed rate *v_f_x_* was maintained along the linear edge of the machined surface in the direction of the *X*-axis. Therefore, the actual feed rate *v_f_* along the tool movement trajectory was variable. This made it possible to compensate for axis tracking errors and the dynamic inertia of the machine tool, which was also observed in the recorded cutting force curves.

(2) $$v_{f\_x}=v_{f\cdot }\sqrt{1+{tan}^{2}\left(\pm \alpha \right)\cdot {cos}^{2}(\frac{x\cdot {tan}^{2}\left(\pm \alpha \right)}{A})}$$

The above relationship takes into account the amplitude *A* and the assumed angle of inclination of the shaping sinusoidal curve *α* (upward + and downward −). In this case, the period *T* is a function of the inclination angle of the shaping sinusoidal curve (see Figure 1).

## 3. Results and Discussion

### 3.1. Reference Tests—Models and Analysis

**Figure 3:**
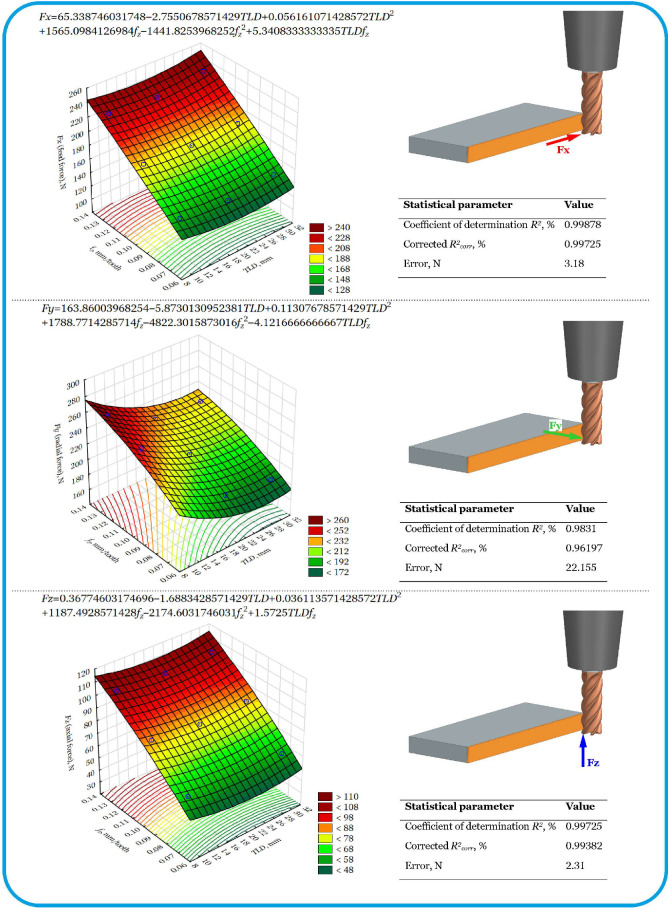
Models of cutting force components for reference cenventional linear milling in the amplitude range *A* = 30 mm.

Cutting force models in conventional linear side milling, used as a reference, are shown in Figure 3.

The increase in cutting force *Fx* (feed force) is mainly influenced by the feed per tooth *f_z_*. This increase shows an almost linear trend and correlates positively with *f_z_*, reaching its maximum value for the highest value of the feed. It has been shown that the effect of the level of displacement of the tool along its axis is nonlinear. The *Fx* force reaches its lowest values in the middle range of the functional length *LF* of the tool, that is, for the second level (see Figure 1). Based on the developed model, it was found that there is an interaction between the input variables and the force *Fx*, but it is insignificant. The model showed very good agreement with the data, as evidenced by the corrected coefficient of determination of 99.75%.

The feed per tooth *f_z_* was found to have a dominant influence on the radial force *Fy*. This effect is nonlinear—the value of force *Fy* increases, reaches a maximum, and then decreases. In turn, the impact of the level of displacement of the tool along its axis is moderate. The force *Fy*, as in the case of *Fx*, reaches its lowest values for the second level (see Figure 1). It was found that the interaction between the input variables and the force *Fy* is significant. The reduction in the force *Fy* occurs for combinations of larger values of tool displacement along its axis *TLD* with smaller values of feed per tooth *f_z_*.

The model was found to be in good agreement with the data, as evidenced by the corrected coefficient of determination of 96.19%. This may be due to the impact of the radial run-out of the cutting edges, exacerbated by their cyclical operation, resulting from the rotation of the tool around its own axis.

In turn, for the axial force *Fz*, a nonlinear effect of feed per tooth was observed. At low values of the feed per tooth *f_z_* parameter, the force *Fz* increases and then decreases. It has been observed that the force *Fz* decreases with increasing displacement of the tool along its axis TLD. The model showed very good agreement with the data, as evidenced by the corrected coefficient of determination of 99.38%.

### 3.2. Fundamental Tests—Models and Analysis

**Figure 4:**
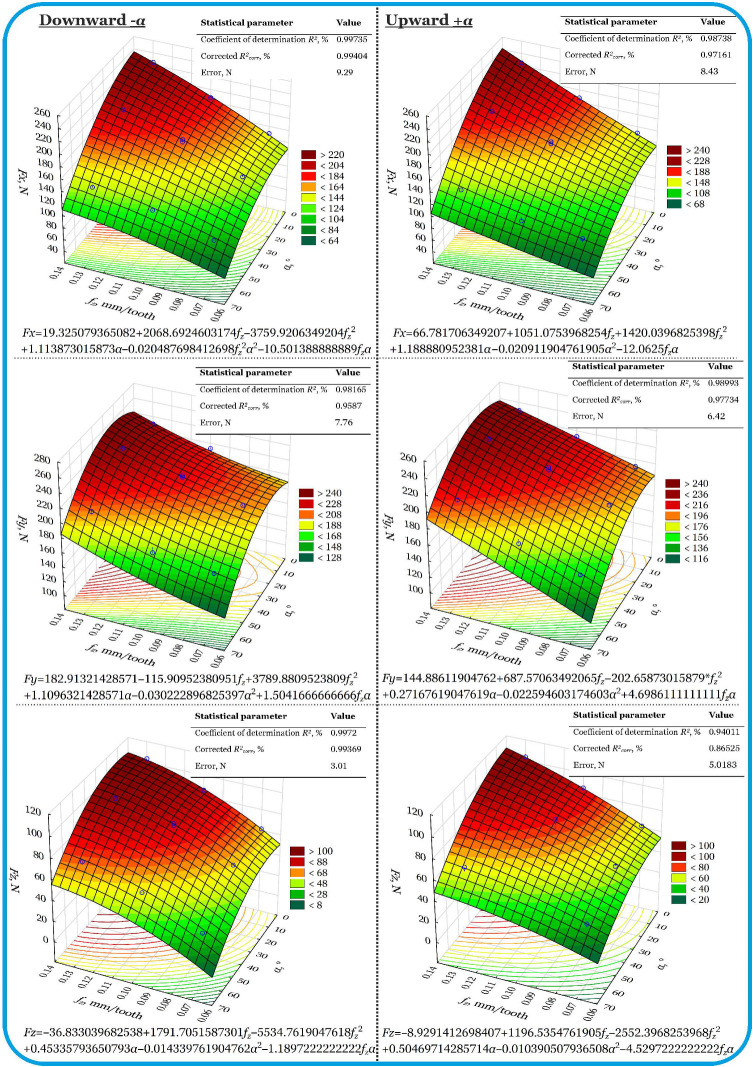
Models of cutting force components for unconventional oscillating milling in the amplitude range *A* = 30 mm.

Cutting force models for unconventional side oscillation milling are shown in Figure 4. Modeling and analysis were performed separately for the downward and upward motion of the tool along a sinusoidal trajectory. The feed rate vector *v_f_* was aligned with the tool path, as is generally accepted (see Figure 1).

#### 3.2.1. Downward (α−)

It has been found that the force *Fx* is most strongly influenced by the feed per tooth *f_z_*. This is a nonlinear effect, with the force *Fx* increasing and then decreasing. The positive influence of angle *α* on force *Fx* was also demonstrated. In addition, the interaction of input variables leads to a significant increase in *Fx*. The model showed very good agreement with the data, as evidenced by the corrected coefficient of determination of 99.404%.

In the case of radial force *Fy*, it was found that the effect of feed per tooth on *Fy* is nonlinear. The minimum values of the force *Fy* were recorded for low feed rates, while for higher feed rates, the force *Fy* increased more rapidly. The value of the force *Fy* correlates positively with angle *α*, i.e., as the value of the angle *α* increases, the force *Fy* also increases. The influence of input variable interactions on force *Fy* is insignificant. The model showed very good agreement with the data, as evidenced by the corrected coefficient of determination of 99.598%.

However, in the case of the axial force *Fz*, it was found that the feed per tooth exerts a decisive influence on *Fz*, following a nonlinear trend. The influence of angle α and the effect of the interaction between input variables on *Fz* is insignificant. The model showed very good agreement with the data, as evidenced by the corrected coefficient of determination of 99.369%.

#### 3.2.2. Upward (α+)

It has been found that the feed per tooth *f_z_* has a classic increasing effect on the force *Fx*. A positive influence of angle α on the force *Fx* was also demonstrated, as in the case of downward movement. The interaction between input variables has a very significant effect on the force *Fx* in this case. This causes a drastic increase in the feed force *Fx*. The model showed good agreement with the data, as evidenced by the corrected coefficient of determination of 97.61%.

In the case of radial force *Fy*, it was found that the effect of feed per tooth on *Fy* increases in a nonlinear manner. The effect of angle *α* on force *Fy* is insignificant. In turn, the influence of the interaction of the input variables on the force *Fy* is significant, especially for larger values of the angle *α*. This differs from the downward movement, which may be due to the geometry of the tool. The model was found to be in good agreement with the data, as evidenced by the corrected coefficient of determination of 96.05%.

However, in the case of the axial force *Fz*, it was found that the feed per tooth has a decisive influence on *Fz*_,_ which follows a parabolic trend. A positive influence of angle *α* on force *Fz* has been demonstrated, especially at high values of the toolpath inclination angle. The interaction between input variables has a very significant effect on *Fz* and has a damping effect, i.e., it can contribute to a reduction in the axial force *Fz*. The model was found to be in good agreement with the data, as evidenced by the corrected coefficient of determination of 94.61%.

Compared to linear tests, oscillatory milling (both upward and downward) shows greater sensitivity of cutting forces to the interaction between the inclination angle *α* and the feed per tooth *f_z_*. In the case of forces *Fx* and *Fy*, the sinusoidal trajectory of the tool movement significantly increases the complexity of the force distribution, especially at higher values of angle *α* and higher feed rates. The force *Fz* remains the most stable and shows a similar pattern to that in linear tests, although with visible fluctuations as the angle *α* increases. *RSM* models for side oscillating milling have a higher fitting accuracy (*R*^2^), which results from the generally assumed feed rate along the toolpath trajectory. Therefore, it is necessary to compensate for the cutting forces by optimizing the feed rate value with respect to the straight edge of the machined surface.

The results obtained were referred to in [17], as the only currently available study that is most similar in terms of the research conducted. Based on the results of this study and study [17], it can be concluded that the tool paths influence all of the components of the cutting force. There is a decreasing trend when a sinusoidal path is used when compared to the linear path. In general, two scenarios are conceivable. Firstly, there is the possibility of an enhancement in comparison to the linear trajectory. However, these alterations may be precluded by the correlation between the dimensions of the feed and the *TLD* parameter.

The cutting force, responsible for the major portion of the cutting force in machining, is influenced by the workpiece material, tool material, tool geometry, and cutting conditions [9,13,22,39]. In principle, the cutting force is contingent upon the specific cutting force and cutting cross section at the tool–chip interface. This interface is associated with the undeformed chip width and the undeformed cutting thickness, or width of cut and feed per tooth. It can thus be concluded that factors which alter specific cutting forces and/or cutting cross-sections have the capacity to affect the cutting forces, particularly in the context of the sinusoidal tool trajectory.

It is noteworthy that two distinct movements should be considered. Firstly, there is a programmed feed movement; secondly, there is an additional relative movement resulting from the tool’s helical structure. It has been demonstrated that the sinusoidal path increases the effective feed in the perpendicular direction of the programmed feed. However, it has also been established that the maximum removed material thickness remains constant. Consequently, this increase in feed results in a reduction in the specific cutting force, thereby causing the forces to become smaller than those in a linear path. However, should the largest increase occur in the feed, the cutting cross-section effect could exceed the specific cutting force effect. This would result in an increase in the cutting force. This phenomenon has been previously observed in [17]. The following section will describe an attempt to optimize the process.

### 3.3. Fundamental Tests—Compensation

**Figure 5:**
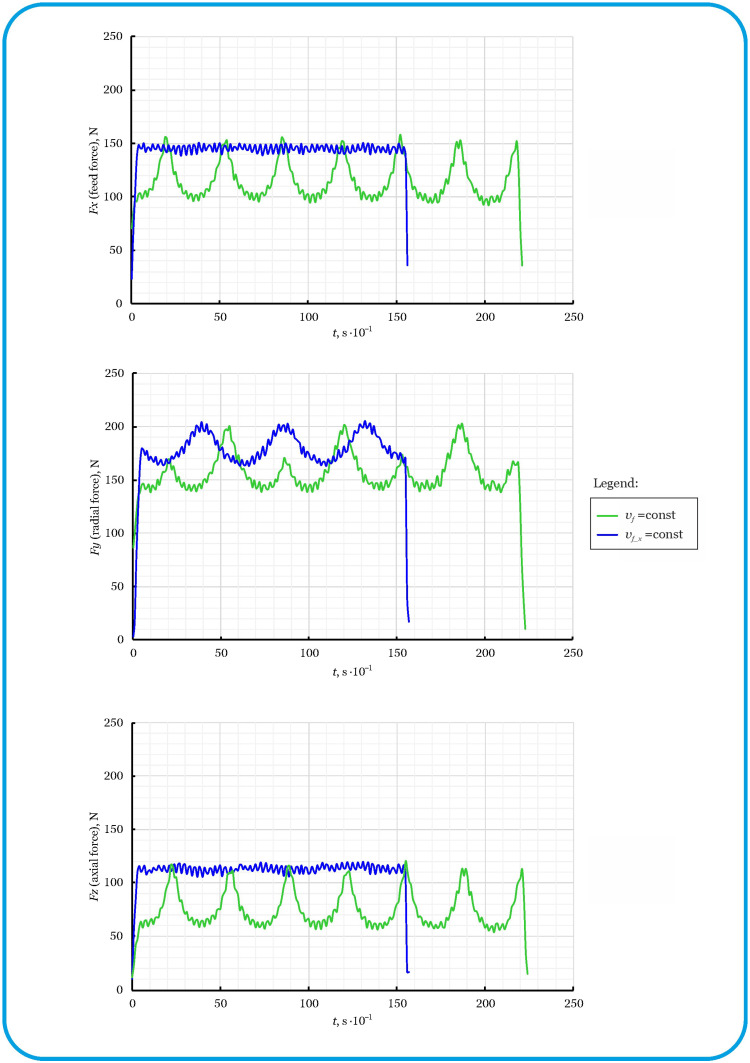
Cutting force curves during oscillation side milling with feed before (*v_f_*) and after (*v_f_x_*) optimization.

As shown by the analysis of the test results, clear differences in the nature of tool loads were observed depending on the feed strategy (linear and sinusoidal with different angles of inclination of the shaping curves). In the above tests, the feed rate *v_f_* was kept constant along the toolpath. Compensation for cutting force fluctuations in side oscillation milling was achieved by rationalizing the feed rate (see relationship (1)) in such a way that the feed rate component *v_f_x_* along the *X*-axis remained constant despite the sinusoidal path of the tool in the *Z*-axis. The cutting force curves for unconventional oscillatory side milling with a constant feed *v_f_* and a constant feed *v_f_x_* (variable feed *v_f_*) are shown in Figure 5.

In the case of the constant feed rate *v_f_* strategy, significant fluctuations in cutting forces were observed in all three axes (*X*, *Y*, *Z*), resulting from the variable feed rate *v_f_x_* component along the sinusoidal toolpath. Sinusoidal motion in the *Z*-axis causes the feed rate of the tool’s feed rate relative to the material along the *X*-axis to be variable, which directly translates into variability in cutting conditions. However, the use of a feed optimization approach, in which the value of *v_f_* changes dynamically depending on the position of the tool on the *Z*-axis, allowed the feed component *v_f_x_* to remain constant and had a clear effect on the stabilization of the cutting forces. This was particularly noticeable in the *X-* and *Y*-axes, where the cutting forces remained almost constant for the strategy with a rationalized feed rate, in contrast to the strongly fluctuating curve with a constant value *v_f_*.

Furthermore, although the cutting depth was constant, the change in the height of contact between the tool and the workpiece caused the cutting forces to change continuously. This results in observable differences in cutting force periods and amplitudes, particularly on the Y-axis, even with strategies that have a rationalized feed rate.

Although the sinusoidal curve of the toolpath was identical in both cases, the strategy with the rationalized feed rate, due to local accelerations of movement, reduced the tool’s travel time along the entire path by approximately 8 s. This phenomenon was reflected in shorter time traces of the compensated cutting forces using a strategy with a rationalized feed rate.

## 4. Conclusions

Experimental and model studies confirm the significant influence of the toolpath geometry, tool level depth (*TLD*), and parameters *f_z_*, *v_f_*, *v_f_x_*, and *α* on the cutting forces. Furthermore, it has been demonstrated that

- Sinusoidal motion (especially upward) introduces strong interactions between the feed and toolpath geometry (angle *α*).
- The feed force *Fx* and the radial force *Fy* are strongly dependent on the angle of inclination *α* of the sinusoidal curve that shapes the side surface of the workpiece—a higher inclination (*α* = 60°) significantly increases the load on the tool, especially at high feed rates.
- The axial force *Fz* is least sensitive to changes in the parameter *α*, but interactions with the feed are significant.
- The RSM models have very high coefficients of determination, most above 99%, which confirms their usefulness for predicting and optimizing the oscillating side milling process.
- In terms of minimizing cutting forces, a low feed rate and moderate angle *α* (e.g., 30°) are recommended; at 60°, the geometric effect and the servo mechanism tracking errors of the machine tool outweigh the technological benefits.

In summary, the use of a variable *v_f_* parameter, which allows the feed rate *v_f_x_* to remain constant, significantly improves the stability of the cutting process. It reduces load variability in each axis, limits dynamic overloads, and increases machining efficiency (by approximately 30%). It may also contribute to extending the tool life and improving the quality of the machined surface, which will be the subject of further research in the above-mentioned area.

## Data Availability

The original contributions presented in the study are included in the article, further inquiries can be directed to the corresponding author.
